# First Morpho-Functional Assessment of Immature Stages of *Pelecocera* Species (Diptera: Syrphidae) Feeding on False Truffles

**DOI:** 10.3390/insects15030191

**Published:** 2024-03-13

**Authors:** José J. Orengo-Green, M. Ángeles Marcos-García, Leif Bloss Carstensen, Antonio Ricarte

**Affiliations:** 1 Research Institute CIBIO (Centro Iberoamericano de la Biodiversidad), University of Alicante, San Vicente del Raspeig, 03690 Alicante, Spain; marcos@ua.es (M.Á.M.-G.); antonio.ricarte@ua.es (A.R.); 2 Independent Researcher, Stenvangen 4, 8850 Bjerringbro, Denmark; lasiopa@bknet.dk

**Keywords:** chaetotaxy, Denmark, hoverfly, larva, mycophagous, *Pelecocera lugubris*, *Pelecocera tricincta*, puparium, *Rhizopogon*

## Abstract

**Simple Summary:**

The scarce genus of *Pelecocera* Meigen, 1822 (Diptera: Syrphidae) from the Holarctic Region has 14 species described. The taxonomic diagnosis of the immature stages of *Pelecocera* has not been performed; however, *Pelecocera* (*Chamaesyrphus*) *japonica* (Shiraki, 1956) larvae were found feeding on *Rhizopogon roseolus* in Japan. Following the findings in Japan, larvae of *Pelecocera* were collected in Denmark. We here report the first morphological description of the immature stages of *Pelecocera* (*Chamaesyrphus*) *lugubris* and *Pelecocera* (*Pelecocera*) *tricincta*, as well as specific data on their breeding sites. Larvae of both species were collected feeding on *Rhizopogon luteolus* in Denmark. The morphology of immature stages of *P. lugubris* and *P. tricincta* was studied by using both a scanning electron microscope and a stereomicroscope. A taxonomic diagnosis of the immature stages of *Pelecocera* and a taxonomic key are provided to separate them from the larvae of other genera.

**Abstract:**

With 14 species, *Pelecocera* Meigen, 1822 is a scarce and small genus of hoverflies (Diptera: Syrphidae: Rhingiini) from the Holarctic Region. Apart from the finding of larvae of *Pelecocera* (*Chamaesyrphus*) *japonica* (Shiraki, 1956) in fungi in Japan, the larval biology of these hoverflies is virtually unknown. The early stages of all *Pelecocera* species are undescribed. The adults of *Pelecocera* (*Pelecocera*) *tricincta* Meigen, 1822 and *Pelecocera* (*Chamaesyrphus*) *lugubris* Perris, 1839 are found in Palearctic conifer forests with sand dunes. We here report the first morphological evidence of the immature stages of *Pelecocera* (*P. lugubris* and *P. tricincta*), as well as specific data on their breeding sites. Larvae of both species were collected feeding on the hypogean basidiomycete *Rhizopogon luteolus* Fr. & Nordholm, 1817 in Denmark in 2021. The first larval stage and second larval stage of *P. tricincta*, the third larval stage of *P. lugubris*, the anterior respiratory process, and the posterior respiratory process of the puparia of these two species were analyzed and studied using stereomicroscope and scanning electron microscope techniques. The chaetotaxy of the puparium of each species is also described and illustrated. A taxonomic diagnosis of the larvae of the genus *Pelecocera* is proposed to separate them from the larvae of other genera of the tribe.

## 1. Introduction

*Pelecocera* Meigen, 1882 is one of the rarest and smallest genera of hoverflies (Diptera: Syrphidae), with 14 species described from the Holarctic Region [1,2,3,4]. The genus *Pelecocera* belongs to the tribe Rhingiini [5,6], and there is still some controversy regarding its phylogenetic relationships. Some recent works propose *Portevinia* Goffe, 1944 as the sister group of *Pelecocera* [7,8], while others propose *Ferdinandea* Rondani, 1844 [9]. In addition, *Chamaesyrphus* Mik, 1895, is considered a genus [10,11] or a subgenus of *Pelecocera* [12]. This has, however, already been resolved in the work performed by Vujić et al. [7], as it mentions that *Chamaesyrphus* is a subgenus of *Pelecocera* based on morphological and molecular characters.

*Pelecocera* (*Pelecocera*) *tricincta* Meigen, 1822 and *Pelecocera* (*Chamaesyrphus*) *lugubris* Perris, 1839 are two of the eight species occurring in Europe [4]. However, the number of species in Europe remains unclear because a worldwide revision of the genus is needed to resolve all the taxonomic doubts that exist at the species level. *Pelecocera tricincta* can be found from the Iberian Peninsula to Siberia, including the Caucasus [12], while *P. lugubris* is present from Portugal to Scandinavia [4]. The distribution of range of both species might be wider than currently known, especially that of *P. lugubris*, due to inaccurate knowledge of their adult phenology and inconspicuous presence in the field.

The adults of *Pelecocera* can be distinguished from other genera by their narrow black thorax, black with yellow abdominal spots, small size (<10 mm), thickness and position of the arista (hair-like and near the base of the basoflagellomere in *Pelecocera* (*Chamaesyrphus*) and thick and near the tip of the basoflagellomere in *Pelecocera* (*Pelecocera*), bare metasternum, and straight vein R_4+5_ and crossvein r-m before the middle of the cell dm [1,13].

Due to the almost total absence of data on the larval morphology, there is no taxonomic diagnosis of the immature stages of *Pelecocera*, and there are no keys to separate larvae of this genus from those of other hoverfly genera. Only the morphological sculpture of the egg of *Pelecocera* (*Chamaesyrphus*) *lusitanica* (Mik, 1898) and *P. tricincta* was described by Kuznetzov [14].

Okada et al. [11] reported that the oviposition sites of *Pelecocera* (*Chamaesyrphus*) *japonica* (Shiraki, 1956) in Japan are the maturing fruiting bodies of the fungi *Rhizopogon roseolus* (Corda) Th. Fr., 1909 and *Rhizopogon luteolus* Fr. & Nordholm, 1817, from which larvae were collected. This discovery led to the assumption that the larvae of *Pelecocera* are mycophagous, but more fieldwork is required to understand the trophic regimes of the *Pelecocera* species. However, Speight [12] mentions that *Pelecocera caledonica* (Collin, 1940), *P. lusitanica*, and *Pelecocera* (*Chamaesyrphus*) *scaevoides* (Fallén, 1817) are apparently phytophagous.

This study aims to provide the first evidence of the larval morphology of *Pelecocera* hoverflies worldwide, as well as further data on their breeding sites in Europe from the findings of two species, *P. tricincta* and *P. lugubris*. In addition, a taxonomic diagnosis for the larvae of the *Pelecocera* species and a comparison between the immature stages of related genera are provided.

## 2. Materials and Methods

### 2.1. Examined Material and Adult/Larva Identification

**Figure 1:**
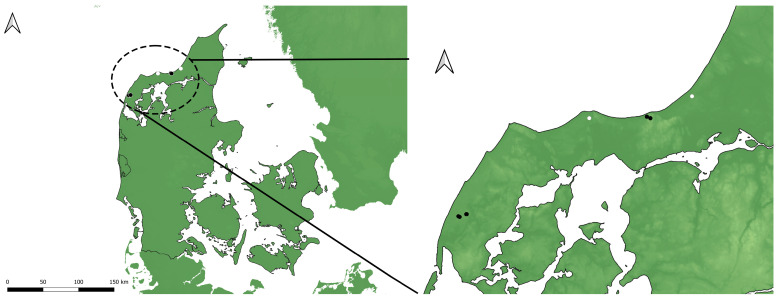
Localities where *Pelecocera* larvae were found in Denmark. Legend: white circle indicates *Pelecocera (Chamaesyrphus) lugubris*; black circle indicates *Pelecocera (Pelecocera) tricincta* and *P. lugubris*.

**Figure 2:**
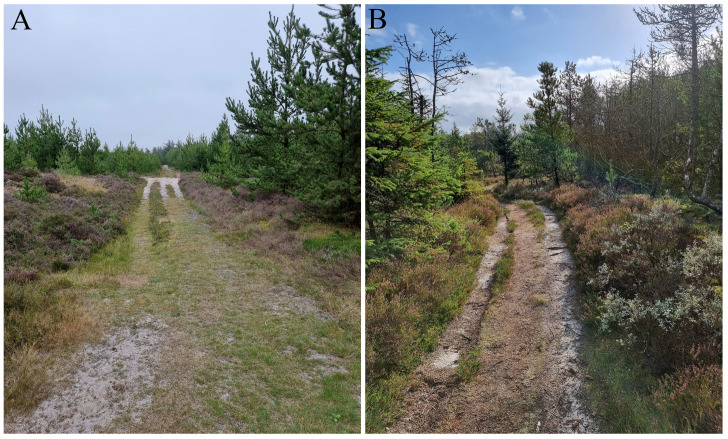
Examples of sampling area: (**A**) Hvidbjerg Klitplantage; (**B**) Lild Klitplantage (Photos: Leif Bloss Carstensen).

**Figure 3:**
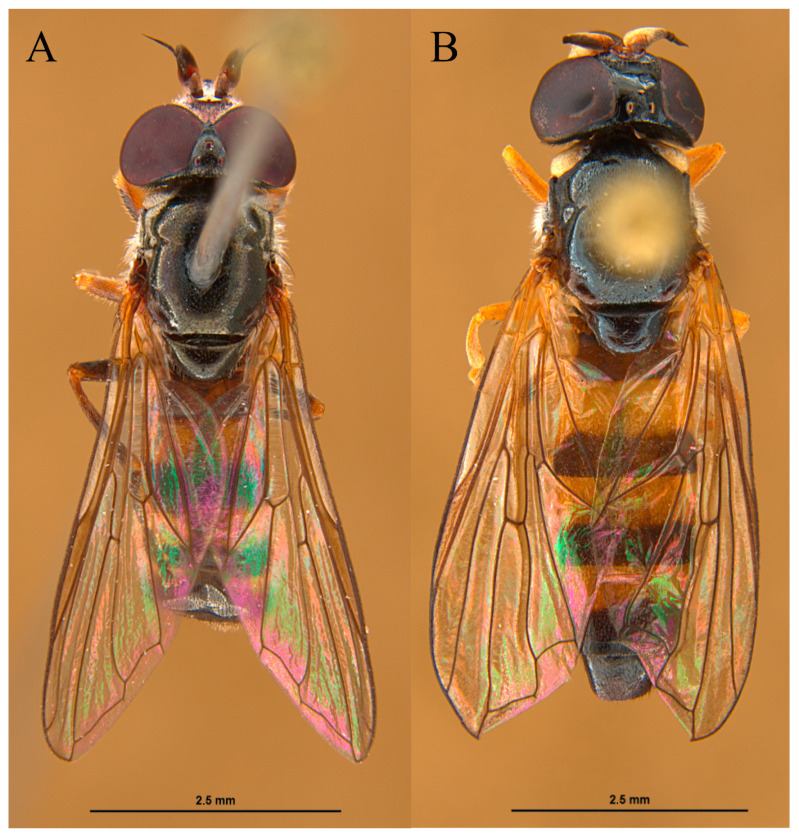
*Pelecocera* adults reared from larvae collected in Hvidbjerg Klitplantage and Svinkløv Klitplantage, Denmark: (**A**) Male of *Pelecocera (Chamaesyrphus) lugubris*; (**B**) Female of *Pelecocera (Pelecocera) tricincta*.

Fruiting bodies of *R. luteolus* with both species (*P. lugubris* (*n* = 8) and *P. tricincta* (*n* = 9)) were found on several locations in Denmark, such as Hvidbjerg Klitplantage (56.8619, 8.3325) and Svinkløv Klitplantage (57.1420, 9.3033) (Figure 1) by Leif Bloss Carstensen in September 2021. The “Klitplantage” is a dune plantation made to prevent sand drifts. Most of the trees present on both sites are conifers, especially *Pinus mugo* Turra, 1764 (Figure 2). The fruiting bodies were at ground level up to ten meters from pine trees. Only a few specimens of *l1* were checked for larvae on site, and not all were infested. Some fruiting bodies and the sand underneath had over 30 larvae (Leif Bloss Carstensen, pers. com.). The larvae were reared in several small plastic containers with one or few fruiting bodies from the same location. The containers were stored in a cupboard in a partially shaped part of a carport at environmental temperature (−15 °C to 32 °C) (September 2021–September 2022). The containers were checked daily to record changes in the immature development. Three (one first stage (L1) and two second stage (L2)) larvae of *P. tricincta* and one third stage (L3) larva of *P. lugubris* were preserved in 70% alcohol, and the rest were reared for adult identification. Six larvae of *P. tricincta* and seven larvae of *P. lugubris* pupated and five adults of each species emerged from these puparia (Figure 3). The match between the larvae and the pupae was based on the features of the posterior respiratory process (PRP). Adults were identified using the taxonomic key of Lair et al. [4]. Examined species were deposited at the CEUA-CIBIO collection, University of Alicante, Spain.

### 2.2. Sample Preparation and Study

The pupae were cleaned in an ultrasonic bath for 10 min and brushed to remove any dirt. The head skeleton was removed from one L2 larva of *P. tricincta* by soaking it in hot 10% KOH for 5 min, and it was examined in glycerin. General features of the larva, puparium, and head skeleton were observed under a Leica M205 C binocular stereomicroscope (Leica Camera AG, Wetzlar, Germany). The asterisk (*) in Section 3.4 indicates the most distinguishable characters. The length/width of the larva/puparium were measured at their maxima, with the width always in the abdomen (Figure 10). The measurements of pupal spiracles were length from the base to the apex, width at the maximum point in the middle, and space between the apices of the pupal spiracles (Figure 12E). For the PRP, the width was measured at the transverse ridge and the length above/below the transverse ridge. Photos were produced as stacks of individual images made with a camera (Leica DMC 5400, Leica Camera AG, Wetzlar, Germany) attached to a binocular stereomicroscope (Leica M205 C). Stacks were made in Leica Application Suite Las X^®^, v.4.12.0, Leica Microsystems, Wetzlar, Germany. The drawings were made from printed photos. The colors on the head skeleton drawings indicate the level of sclerotization (lighter = less, dark = heavy). The distribution map of the collected Pelecocera larvae in Denmark was produced with the software QGis 3.32 [15]. For a more detailed description of the anterior respiratory process (ARP), pupal spiracles, and PRP, a scanning electron microscope (SEM) was used. One puparium and one larva were mounted on aluminum stubs with double-sided adhesive carbon tape. The samples were imaged with a Jeol JSM-IT500HR SEM (JEOL Ltd., Tokyo, Japan) in variable pressure mode to be able to recover the material.

### 2.3. Morphological Terminology

The terminology used for the description of larvae and puparia follows Rotheray [16]. For each body segment, sensilla were numbered in the dorsoventral direction [17]. A superscript (^A1…^) is used to indicate in which body segment a sensilla is located (e.g., 1^A1^—first sensilla of the first abdominal segment). The terminology used for the head skeleton follows Hartley [18]. A compilation of abbreviations for morphological features used in this publication is shown in Table 1.

## 3. Results

### 3.1. Shared Descriptions of the Larvae/Puparia of Pelecocera (Pelecocera) tricincta and Pelecocera (Chamaesyrphus) lugubris

**Figure 4:**
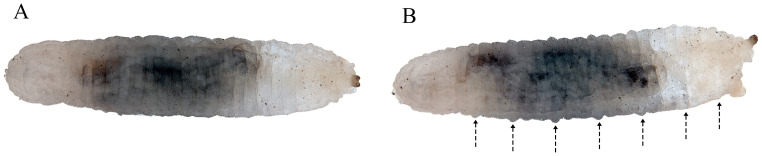
Second stage larva (L2) of *Pelecocera (Pelecocera) tricincta*: (**A**) Dorsal view; (**B**) Lateral view. Dash arrows indicate locomotory prominences.

Vermiform larva with the eighth abdominal segment (=anal segment) small and abruptly truncated with three pairs of well-developed lappets (Figure 4 and Figure 13). Prothorax with a pair of well-developed antenna-maxillary organs slightly sclerotized, mounted on a fleshy projection (Figure 6). Dorsal laterally of the prothorax with a pair of small, sclerotized ARP with wrinkled surface basally, smooth at the rest, and the apex with a tip with two spiracular openings (Figure 7). A pair of developed locomotory prominences without crochets in the mesothorax and from the first to the seventh abdominal segments (Figure 4B and Figure 13B). Outline of the PRP in dorsal view M-shaped PRP; tapering toward the apex in the lateral view but swollen above the transverse ridge (Figure 8). Spiracular plate with four pairs of long interspiracular setae, three pairs of spiracular openings, a pair of ecdysial scars, and a pair of perispiracular glands (Figure 9). Chaetotaxy (Figure 10): all observed sensilla-bearing setae. Prothorax: dorsal side with four pairs of sensilla (1^Pt^–4^Pt^), lateral side with three pairs (5^Pt^–7^Pt^) and ventral side with one pair (8^Pt^). Mesothorax: dorsally with three pairs of sensilla (1^Ms^–3^Ms^), laterally with two pairs (4^Ms^–5^Ms^), and ventrally with three pairs (6^Ms^–8^Ms^). Metathorax: dorsally with three pairs (1^Mt^–3^Mt^), laterally with two pairs (4^Mt^–5^Mt^), and ventrally with three pairs (6^Mt^–8^Mt^). Abdomen: from the first to the seventh abdominal segments dorsally with three pairs of sensilla (1^A1–7^–3^A1–7^), laterally with four pairs (4^A1–7^–7^A1–7^), and ventrally with three pairs (8^A1–7^–10^A1–7^). Anal segment with two pairs of sensilla (1^A8^–2^A8^) at the tip of the dorsal lappet; two pairs (3^A8^–4^A8^) at the lateral side of the PRP; two pairs (5^A8^–6^A8^) at the tip of the ventral PRP lappet; one pair (7^A8^) at the tip of the ventral lappet; and three pairs (8^A8^–10^A8^) ventrally.

### 3.2. Immature Stages of Pelecocera (Pelecocera) tricincta

#### 3.2.1. L1 Larva

**Description**. Length: 3.35 mm; height: 0.65 mm; and width: 0.71 mm (*n* = 1). Whitish transparent color. *PRP*: yellowish, with a noticeable transverse ridge.

#### 3.2.2. L2 Larva (Figure 4)

**Figure 5:**
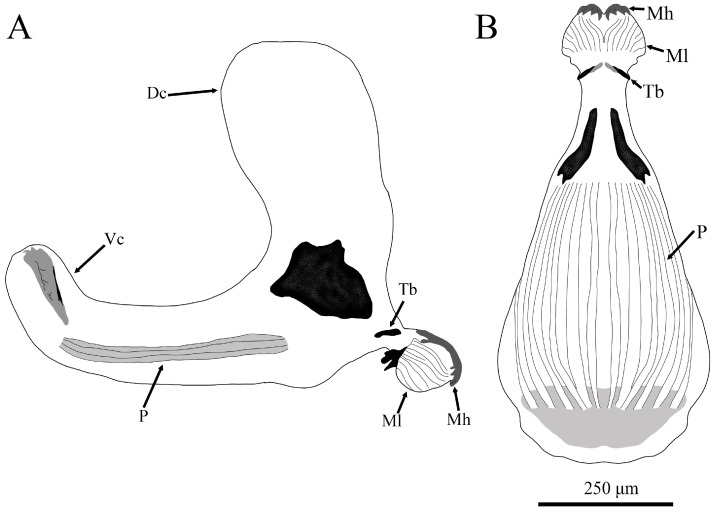
Drawing of the head skeleton of a second stage larva (L2) of *Pelecocera (Pelecocera) tricincta*: (**A**) Lateral view; (**B**) Ventral view. Legend: Dc, dorsal cornu; Mh, mouth hook; Ml, mandibular lobe; P, pharyngeal ridges; Tb, tentorial bar; Vc, ventral cornu.

**Description**. Length: 6.39–6.45 mm; height: 1.2–1.4 mm; and width: 1.29–1.59 mm (*n* = 2). Whitish transparent color. *Head skeleton* (Figure 5): Serrated mouth hooks slightly sclerotized which do not protrude from the mouth; fleshy mandibular lobes; tentorial bar small and highly sclerotized with some parts less sclerotized (only appreciable at ventral view Figure 5B); dorsal cornu of same length as ventral cornu; pharyngeal ridges of same length as ventral cornu. *PRP*: yellowish, with a noticeable transverse ridge.

**Figure 6:**
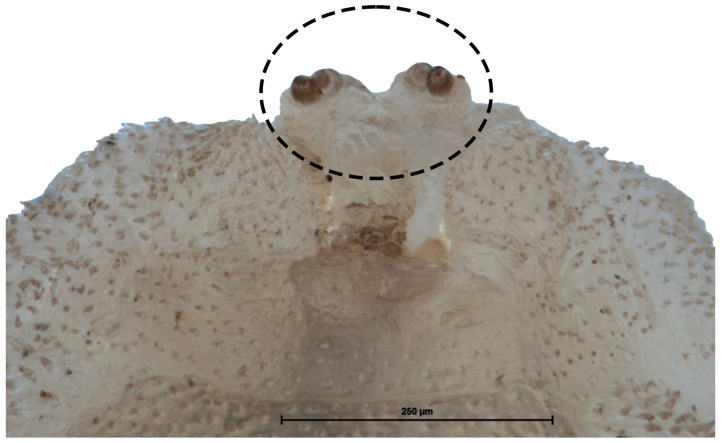
Prothorax ventral view of a second stage larva (L2) larva of *Pelecocera (Pelecocera) tricincta*. A dash circle indicates the antenna-maxillary organs.

**Figure 7:**
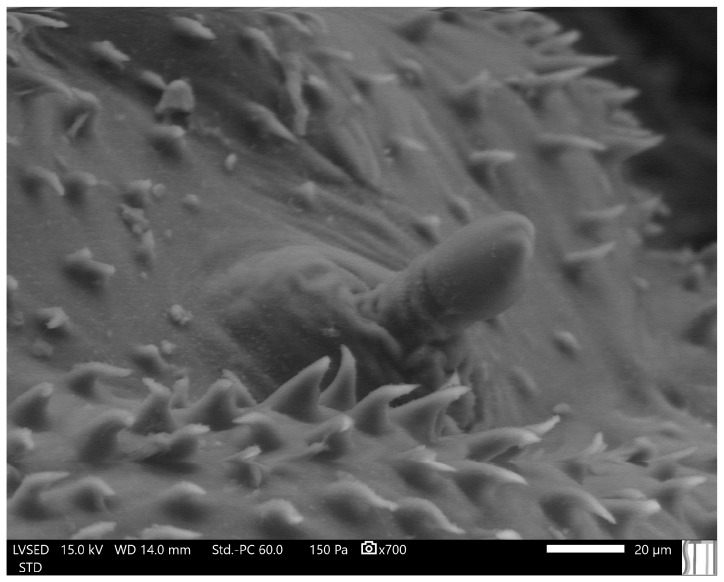
Anterior respiratory process of a second stage larva (L2) larva of *Pelecocera (Pelecocera) tricincta*, dorsal-lateral view.

**Figure 8:**
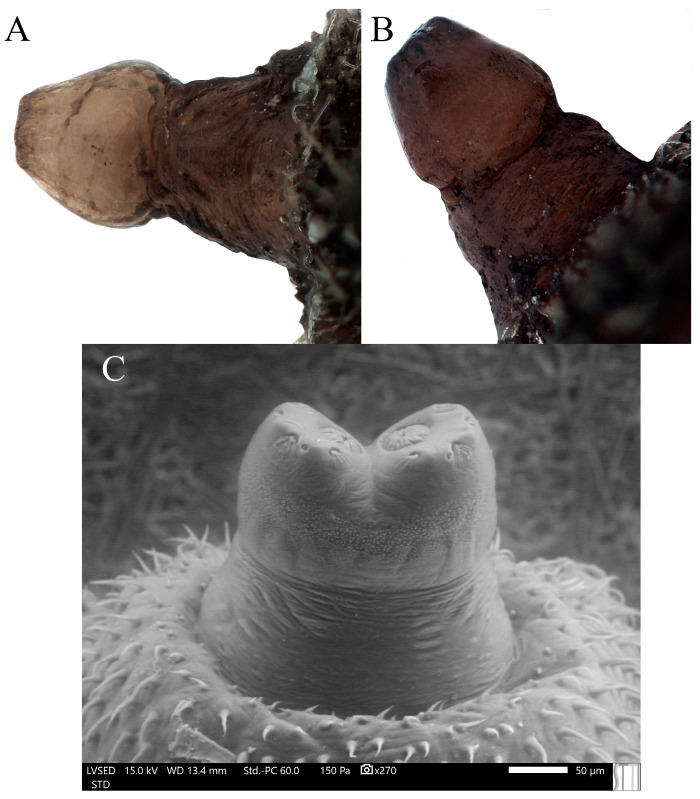
Posterior respiratory process of a second stage larva (L2) and puparium of *Pelecocera* species: (**A**) Puparium of *Pelecocera (Pelecocera) tricincta*, lateral view (stereomicroscope image); (**B**) Puparium of *Pelecocera (Chamaesyrphus) lugubris*, lateral view (stereomicroscope image); (**C**) L2 of *Pelecocera (Pelecocera) tricincta*, dorsal view (SEM) image.

**Figure 9:**
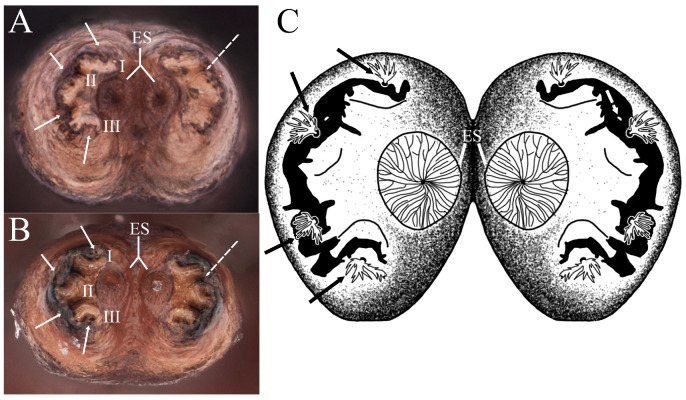
Posterior respiratory process of pupae of *Pelecocera species*: (**A**) *Pelecocera (Pelecocera) tricincta*, polar view (stereomicroscope image); (**B**) *Pelecocera (Chamaesyrphus) lugubris*, polar view (stereomicroscope image); (**C**) *Pelecocera (Pelecocera) tricincta*, polar view (drawing). Legend: Interspiracular setae indicated with an arrow; perispiracular gland indicated with a dash arrow; I, II, and III spiracular openings; ES, ecdysial scar.

**Figure 10:**
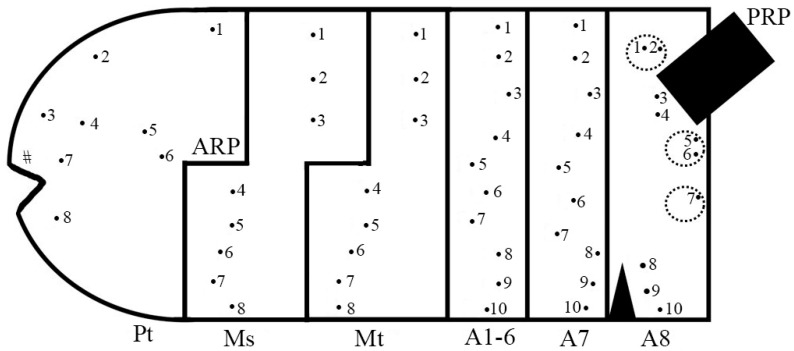
Chaetotaxy map of *Pelecocera* species showing the number and relative positions of the body sensilla. Legend: Pt, prothorax; Ms, mesothorax; Mt, metathorax, A1–A8, abdominal segments; ARP, anterior respiratory process; PRP, posterior respiratory process; #, antenna-maxillary organs; •, sensilla with seta; a dash circle indicates a lappet position.

#### 3.2.3. Puparium of *Pelecocera* (*Pelecocera*) *tricincta* (Figure 11)

**Figure 11:**
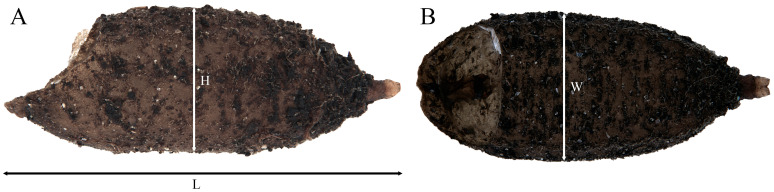
Puparium of *Pelecocera (Pelecocera) tricincta*: (**A**) Lateral view; (**B**) Dorsal view. Legend: H, height; L, length; W, width.

**Figure 12:**
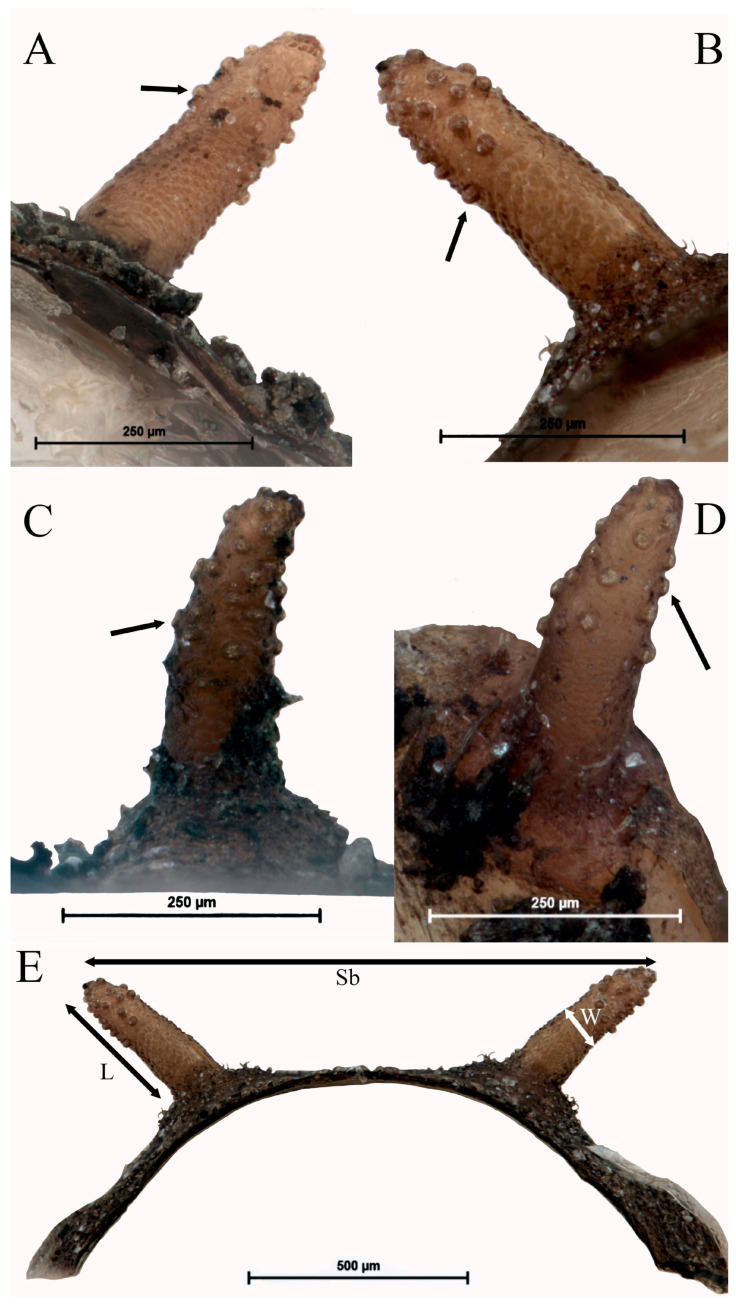
Pupal spiracles of *Pelecocera (Pelecocera) tricincta* and *Pelecocera (Chamaesyrphus) lugubris*: (**A**) *Pelecocera tricincta*, posterior side; (**B**) *Pelecocera tricincta*, anterior side; (**C**) *Pelecocera lugubris*, posterior side; (**D**) *Pelecocera lugubris*, anterior side; (**E**) Indication of distance measured for the descriptions. Legend: L, length; Sb, space between the pupal spiracle tips; W, width. Tubercles indicated with an arrow.

**Description.** Length: 4.79–5.7 mm; height: 1.78–2.14 mm; and width: 1.82–2.23 mm (*n* = 6). Elliptic form with anterior part wider and flat ventrally. Posterior end straight (Figure 11). *Pupal spiracles* (Figure 12A,B): Length: 0.34–0.38 mm; width: 0.1–0.11 mm; and space between the pupal spiracle tips: 1.04–1.21 mm (*n* = 4). Light cream color: cylindrical, tapering apically with a pointed protrusion or rounded spiny tip; yellow tubercles with 3–5 openings. Posterior side with granulated surface at the base and in the apex, and tubercles located at the center. Anterior side with heavily granulated surface at the base and less at the tip; tubercle observed from the upper half to the apex. *PRP*: yellowish color, with a noticeable transverse ridge (Figure 8A). Length above the transverse ridge: 0.18–0.21 mm; length below the transverse ridge: 0.18–0.22 mm; and width at the transverse ridge: 0.25–0.29 mm (*n* = 6).

### 3.3. Immature Stages of Pelecocera (Pelecocera) lugubris

#### 3.3.1. L3 Larva (Figure 13)

**Description**. Length: 8.59 mm; height: 1.76 mm; and width: 2.59 mm (*n* = 1). Dark brown color. *PRP*: Dark brown, with a conspicuous transverse ridge. Length above the transverse ridge: 0.22 mm; length below the transverse ridge: not visible; and width at the transverse ridge: 0.24 mm (*n* = 1).

**Figure 13:**
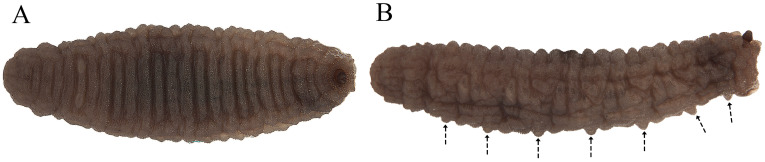
Third stage larva (L3) of *Pelecocera (Chamaesyrphus) lugubris*: (**A**) Dorsal view; (**B**) Lateral view. Dash arrows indicate locomotory prominences.

#### 3.3.2. Puparium of *Pelecocera* (*Chamaesyrphus*) *lugubris* (Figure 14)

**Figure 14:**
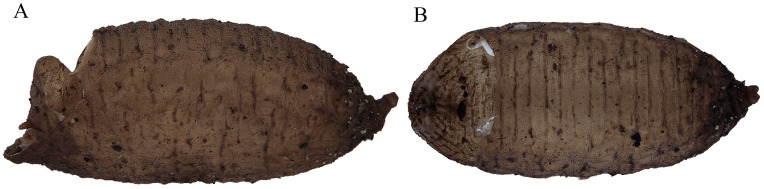
Puparium of *Pelecocera (Chamaesyrphus) lugubris*: (**A**) Lateral view; (**B**) Dorsal view.

**Description.** Length: 4.68–7.2 mm; height: 1.57–2.57 mm; and width: 1.94–2.71 mm (*n* = 7). Elliptic shape with anterior part wider and flat ventrally. Posterior end almost upright (Figure 14). *Pupal spiracles*: Length: 0.16–0.29 mm; width: 0.07–0.09 mm; and space between the pupal spiracle tips: 0.81–1.5 mm (*n* = 5). Dark brown at the base and light brown on the rest (Figure 12C,D); cylindrical tapering toward the apex, with a spiky protuberance at the tip. Tubercles with 3–6 openings located at the top half. Posterior side and anterior side heavily granulated basally; the rest of the surface covered with tubercles. *PRP*: Dark brown color, with conspicuous transverse ridge (Figure 8B). Length above the transverse ridge: 0.15–0.22 mm; length below the transverse ridge: 0.13–0.21 mm; and width at the transverse ridge: 0.26–0.32 mm (*n* = 7).

### 3.4. Immature Stages of Pelecocera: Taxonomic Diagnosis

Mesothorax and first seven abdominal segments with well-developed crochetless locomotory prominences; outline of the PRP in the dorsal view M-shaped PRP* (Figure 8C); eighth abdominal segment small and abruptly truncated posteriorly. In Table 2, we can see a comparison between the considered sister groups of *Pelecocera*.

As a result of our study, the diagnosis of the larvae of *Pelecocera* will be added to the taxonomic key of syrphid larvae of Thompson and Rotheray [26] in step 10, and it will be modified as follows:

10. Body with posterior end with sensilla born on black, stick-like projections; body covered with upright spike-like setae………………………………………***Rhingia* Scopoli, 1763**

10. Body with posterior end with sensilla born on short, conical, and fleshy projections; body covered with short, flattened, and fleshy setae…………………………………………11

11. PRP: M-shaped in dorsal view; eighth abdominal segment small and abruptly truncated……………………………………………………………………***Pelecocera* Meigen, 1822**

11. PRP: Short and slightly constricted in the middle; eighth abdominal segment particularly truncated……………………………………………………***Ferdinandea* Rondani, 1844**

### 3.5. Taxonomic Key for the Immature Stages of the Rhingiini Tribe

1. Mouth hooks not protruding from the mouth………………………………………………2

Mouth hooks protruding from the mouth…………………………………………………4

2. Body with posterior end with sensilla born on black, stick-like projections………***Rhingia***

Body with posterior end with sensilla born on short, conical, and fleshy projections……3

3. PRP: M-shaped in dorsal view; eighth abdominal segment small and abruptly truncated……………………………………………………………………………………***Pelecocera***

PRP: Short and slightly constricted in the middle in dorsal view; eighth abdominal segment particularly truncated………………………………………………………***Ferdinandea***

4. Eighth abdominal segment ends in flattened disc; anus parallel to longitudinal axis of the body………………………………………………………………………………***Portevinia***

Eighth abdominal segment tapering toward the tip; anus transverse to longitudinal axis of the body……………………………………………………………………………***Cheilosia* Meigen, 1838**

## 4. Discussion

Rotheray [27] mentions that all taxonomic keys for immature stages of hoverflies are provisional because there are many genera with undescribed larvae or some have few specimens available for description. In fact, in this work, we are adding the diagnosis of the genus *Pelecocera* to the general knowledge of the early stages of the hoverfly. According to the assessment of the characters states performed, the shape of the PRP appears to be one of the useful characters for this genus.

The immature stages of *Pelecocera* (*Pelecocera*) *tricincta* and *Pelecocera* (*Chamaesyrphus*) *lugubris* shared many features supporting the fact that they are two subgenera instead of two different genera as mentioned in Vujić et al. [7]. Currently, the only way to distinguish these two subgenera is by the color of the PRP; that of *P. tricincta* is yellowish (Figure 8A), and that of *P. lugubris* is dark brown (Figure 8B). This feature must be taken with caution, as more immature stages of *Pelecocera* are found, and this difference may change.

Okada et al. [11] found the larvae of *P. japonica* feeding and developing in *R. luteolus* and *R. roseolus*. They did not describe the larvae but confirmed that *Pelecocera* larvae are true mycophagous, through a gut content analysis, which found undamaged spores of *Rhizopogon*. This is the same as observed with *P. tricincta* and *P. lugubris*, as both species were observed developing on fresh and rotten *R. luteolus*. This implies that the host fungi of the *Pelecocera* species probably are species of *Rhizopogon*, a genus of ectomycorrhizal basidiomycetes, which form hypogeous sporocarps commonly known as false truffles.

*Rhizopogon* is a worldwide fungus genus with over 100 species that are very specific to pine trees, especially those of the genus *Pinus* (Pinaceae) [28,29]. For this reason, *Rhizopogon* has been used in many works related to applications and research in forestry [30,31]. However, during the last decades, the *R. roseolus* has been declining due to the destruction of its coastal habitat in Japan [32]. This is a very important problem for *Pelecocera* as it is very specific to this fungus genus, as was observed in Okada et al. [11] and in our work. For this reason, it is necessary to improve or develop projects for the conservation of the habitat of these fungi, because if they were to disappear, *Pelecocera* would also disappear.

*Ferdinandea* and *Portevinia*, with two stages and one immature stage described, respectively, are the sister groups of *Pelecocera* [7,9,12]. Even though they are genetically related, larvae of these three genera do not share the same trophic habits and breeding sites: *Ferdinandea* larvae are saprophagous (feeding on tree sap) [27]; *Portevinia* larvae are phytophagous (feeding on *Allium* bulbs) [21], and *Pelecocera* larvae are mycophagous (feeding on *Rhizopogon* fungi). These differences in the ecology of these three genera show the importance of studying the larvae and not only the adults. Another difference is the morphology of the eighth abdominal segment, which is small and abruptly truncated (Figure 4B and Figure 13B) in *P. lugubris* and *P. tricincta*, slightly truncated in *Ferdinandea cuprea* (Scopoli, 1763) and *Ferdinandea fumipennis* Kassebeer, 1999 [19,22,25], and flat in *Portevinia maculata* (Fallén, 1817) [21]. These features are the most useful to differentiate these three genera.

Mycophagy is not very common in the immature stages of syrphids, with *Cheilosia* Meigen, 1833 being the genus with the most species with this food spectrum. Unfortunately, the head skeleton of the mycophagous species is not very well known. According to Rotheray and Gilbert [17], in the head skeleton of mycophages, the mandibular lobes are not fused with the mandibular apodemus, and the mandibles and mandibular lobes are slightly sclerotized. All these features can be observed in the head skeleton of *Pelecocera*, confirming that this genus feeds on fungi. Another feature that can be observed is the presence of pharyngeal ridges, which filter and concentrate the food to gain a higher nutritional value [33]. Pharyngeal ridges are not exclusive to mycophages, as they can be found in the immature stages of saprophages (e.g., *Eumerus* Meigen, 1822) [34] and saproxylic hoverflies (e.g., *Milesia* Latreille, 1804) [35].

With the information provided in this work, it is hoped that more immature stages of other *Pelecocera* species can be found to better understand their larval biology. In addition, future work will be performed to find the egg, L1, and L2 of *P. lugubris* and L3 of *P. tricincta* to have more complete information of the morphology of all immature stages and thus be able to facilitate the precise diagnosis of the immature stages of the genus *Pelecocera*.

## Figures and Tables

**Table 1:** Abbreviations used for morphological features of larvae/puparia.

ARP	Anterior respiratory process	Mh	Mouth hook
Dc	Dorsal cornu	Ml	Mandible lobe
ES	Ecdysial scar	P	Pharyngeal ridge
IS	Interespiracular setae	Pg	Perispiracular gland
L1	First larval stage	PRP	Posterior respiratory process
L2	Second larval stage	Tb	Tentorial bar
L3	Third larval stage	Vc	Ventral cornu
M	Mandible		

**Table 2:** Morphological and biological comparison among Pelecocera, Ferdinandea, and Portevinia.

Characters	*Pelecocera* Meigen, 1822	*Ferdinandea* Rondani, 1844	*Portevinia* Goffe, 1944
PRP outline in dorsal view	M-shaped	Short and slightly constricted in the middle (see Figure 1 in Dušek and Láska [19])	Barrel-shaped (see Figure 6 in Rotheray [20])
PRP color	Yellowish/dark brown	Ochre	Shining black [21]
PRP: pairs of spiracular openings	3	3 (see Figure 44 in Hartley [22])	4 [21]
Eighth abdominal segment in lateral view	Small and abruptly truncated	Particularly truncated [22]	Flat disc form (see Figure 1 in Speight [21])
Pairs of lappets	3	3 [19]	Without lappets [21]
Breeding site	Fruiting bodies of *Rhizopogon* spp. fungi	Sap run from *Acer*, *Aesculus*, *Malus*, *Populus*, *Quercus*, and *Salix* trees [19,23,24,25]	*Allium* bulbs [21]

## Data Availability

The data presented in this study are available on request from the corresponding author.
